# Assessing the Causality between Blood Pressure and Retinal Vascular Caliber through Mendelian Randomisation

**DOI:** 10.1038/srep22031

**Published:** 2016-02-25

**Authors:** Ling-Jun Li, Jiemin Liao, Carol Yim-Lui Cheung, M. Kamran Ikram, Tai E. Shyong, Tien-Yin Wong, Ching-Yu Cheng

**Affiliations:** 1 Singapore Eye Research Institute, Singapore National Eye Centre, Singapore; 2 Duke-NUS Graduate Medical School, Singapore; 3 Department of Medicine, National University of Singapore and National University Health System, Singapore; 4 Department of Ophthalmology and Visual Sciences, The Chinese University of Hong Kong, Hong Kong; 5 Department of Ophthalmology, National University of Singapore and National University Health System, Singapore

## Abstract

We aimed to determine the association between blood pressure (BP) and retinal vascular caliber changes that were free from confounders and reverse causation by using Mendelian randomisation. A total of 6528 participants from a multi-ethnic cohort (Chinese, Malays, and Indians) in Singapore were included in this study. Retinal arteriolar and venular caliber was measured by a semi-automated computer program. Genotyping was done using Illumina 610-quad chips. Meta-analysis of association between BP, and retinal arteriolar and venular caliber across three ethnic groups was performed both in conventional linear regression and Mendelian randomisation framework with a genetic risk score of BP as an instrumental variable. In multiple linear regression models, each 10 mm Hg increase in systolic BP, diastolic BP, and mean arterial BP (MAP) was associated with significant decreases in retinal arteriolar caliber of a 1.4, 3.0, and 2.6 μm, and significant decreases in retinal venular caliber of a 0.6, 0.7, and 0.9 μm, respectively. In a Mendelian randomisation model, only associations between DBP and MAP and retinal arteriolar narrowing remained yet its significance was greatly reduced. Our data showed weak evidence of a causal relationship between elevated BP and retinal arteriolar narrowing.

Microvascular abnormalities, in particular retinal arteriolar narrowing, are consistently associated with elevated blood pressure (BP) in clinical studies^1^,^2^,^3^,^4^. Since the haemodynamic auto-regulation is provoked by persistently elevated BP, vasospasm with its myogenic tone may lead to arteriolar remodelling, which results in increased arteriolar resistance and further decompensation of peripheral BP elevation^5^,^6^,^7^,^8^. However, the role of small veins in such pathophysiological process is still not clear, due to the lack of muscular coating in the venular walls. As a window for studying microvasculature *in vivo*, retinal venular caliber has been investigated on its relation with either elevated BP or incident hypertension, yet the findings are not consistent^2^,^9^,^10^,^11^,^12^. The difference in estimates might lie in a few aspects, such as the nature of the study design, potential selection and information bias (e.g. only taking one-time clinical BP into account yet neglecting masked and white coat hypertension), or environmental confounding factors embedded in the studies themselves that could not be corrected or accounted for completely.

Mendelian randomisation provides a method for assessing the causal nature of exposures with health outcomes or diseases, by using genetic variants as robust proxies (i.e. instrumental variables [IVs]) for environmentally modifiable exposures^13^,^14^,^15^,^16^. As part of the genetic epidemiological methodology, this approach applied in our study is based on three assumptions^14^,^17^. First, genetic variants have been identified to be lifetime exposure to affect BP. Second, the BP variants will not be affected by confounders such as lifestyle, socioeconomic status and environmental risk factors, and reverse causation. Third, the association between BP genotype and retinal vascular caliber is only dependent on BP levels.

Given that multiple genetic variants influencing BP have been repeatedly identified through genome-wide association studies (GWAS) in Europeans and/or Asian population^18^,^19^,^20^,^21^,^22^,^23^, our study aimed to use the genetic variants as IV to determine the potential causal effect of BP on retinal vascular caliber in the Singapore Epidemiology of Eye Diseases (SEED) Study, a multi-ethnic, population-based study of Singaporean adults.

## Results

The demographic and clinical characteristics of the study participants are shown in Table 1. The mean (±standard deviation) age of the study participant was 58.1 ± 10.0 years, and 50.7% were males. Among the middle-to-old age population, 62.5% had hypertension while 23.4% had diabetes at the time they were recruited.

In conventional multiple linear regression models, the relationship between elevated BP and retinal arteriolar narrowing was consistent in both age, gender-, and ethnic group-adjusted model (Model 1, Table 2) and a more sophisticated model additionally adjusting for household income, body mass index (BMI), total cholesterol, blood glucose, blood creatinine level, presence of hypertension, presence of diabetes, smoking history, and alcohol drinking history (Model 2, Table 2). Compared to Model 1, the effect size became smaller after additional confounders were taken into account in Model 2, where each 10 mm Hg increase in systolic blood pressure (SBP), diastolic blood pressure (DBP), and mean arterial pressure (MAP) was significantly associated with a 1.4, 3.0, and 2.6 μm decrease, respectively, in central retinal arteriolar equivalent (CRAE) (all P < 0.001). For central retinal venular equivalent (CRVE), each 10 mmHg increase in SBP, DBP, and MAP was significantly related to a 0.6 μm (P < 0.001), 0.7 μm (P < 0.01), and 0.9 μm (P < 0.001) decrease in Model 2, respectively.

In the Mendelian randomisation model adjusted for age, gender and principal components (PCs) (Model 3, Table 2), each 10 mm Hg increase in DBP and MAP was associated with a 4.5 μm decrease (P = 0.01) in CRAE, and the effect size of MAP on CRAE remained similar (β = −2.6 μm) and was marginally significant (P = 0.05). There was no significant association between SBP and CRAE (β = −1.3 μm, P = 0.10). None of the three BP phenotypes remained significantly associated with CRVE.

The possibility of pleiotropic effect tested insignificant in our study (Table 3). No significant association was found between BP genetic risk scores (GRSs) and traditional confounders. Therefore, we believe that the possibility of pleiotropic effect in our Mendelian randomisation model was minimal and it would not affect our findings.

## Discussion

In this population-based cohort study of multi-ethnic Asians aged 40 years and above, we provided weak evidence of a possible causal effect of elevated BP (DBP and MAP) with retinal arteriolar narrowing but not with retinal venular narrowing, by using a Mendelian randomisation approach with BP genetic risk scores as the instrument variable.

As a widely recognised surrogate for general microcirculature *in vivo*, retinal vascular morphology has been suggested to mirror BP variation in several population-based, cross-sectional epidemiological studies^24^,^25^,^26^. Yet on the contrary, the role of retinal venular caliber is still unknown, evidenced by the inconsistent findings in a number of epidemiological and clinical studies. In cross-sectional studies, the relationship between BP and retinal venular narrowing was reported in Multi-ethnic Study of Atherosclerosis (MESA)^27^, Sydney Children Eye Study (SCES)^28^ and Wisconsin Epidemiological Study of Diabetic Retinopathy (WESDR)^10^ to be a 1.8–2.1 μm decrease in retinal venular caliber with each 10 mm Hg increase in MAP. However, the direction was null in the Beaver Dam Eye Study (BDES)^27^ and Singapore Malay Eye Study (SiMES)^1^, or opposite (2.6 μm increase) in the Atherosclerosis Risk in Communities Study (ARIC)^11^.

The inconsistent findings of retinal venular narrowing and widening correlation with elevated BP might lie in the major drawback of the epidemiologic study design; that is, the presence of measured and unmeasured confounders in observational observation. Controlling for confounders has proven to be difficult when retinal vascular caliber is related to many other factors such as age, gender, lifestyle (smoking and alcohol drinking), and chronic conditions (e.g. type 2 diabetes), which greatly influence BP levels and even hypertension. The drawback of using ordinary linear regression in epidemiological studies is that it generally produces biased (e.g. selection bias and information) and inconsistent estimates with changes to population and study design^13^,^14^,^15^. Associations between modifiable exposures and disease seen in observational epidemiology are sometimes confounded and thus misleading, despite our best efforts to improve the design and analysis of the studies.

Mendelian randomisation is a well-known statistical tool for epi-genetic analysis, akin to a randomised control trial without the longitudinal design. If all assumptions of the Mendelian randomisation model are valid, such bias or systemic errors would be very much prevented compared with running an ordinary linear regression^13^,^14^,^15^. Mendelian randomisation uses genetic polymorphisms (e.g. BP genetic variants) that are known to have effects equivalent to those produced by modifiable exposures (e.g. BP). Associations between genetic variants (e.g. BP genetic risk score) and outcome (e.g. retinal vascular caliber) are generally not confounded by behavioural or environmental exposures^13^. Therefore, cognizant of the limitation in observational association between BP and retinal microvasculature, we adopted the principle of Mendelian randomisation and also used BP single nucleotide polymorphisms (SNPs)-derived BP genetic risk score as an IV.

Our findings of observational and IV estimates on the association between BP and retinal arteriolar caliber were consistent; however, this was not the case for the association between BP and venular caliber. After taking all major confounders into account, each 10 mm Hg increase in SBP, DBP, and MAP was significantly associated with a 1.4, 3.0, and a 2.6 μm decrease (all P values < 0.001) in retinal arteriolar caliber in our conventional linear regression model, respectively, and a relatively larger reduction in retinal arteriolar caliber in Mendelian randomisation as 1.3 μm (P = 0.10), 4.5 μm (P = 0.01), and 2.6 μm (P = 0.05), respectively. For CRVE, each 10 mmHg increase in SBP, DBP, and MAP was significantly related to a 0.6 μm (P < 0.001), 0.7 μm (P < 0.01), and 0.9 μm (P < 0.001) decrease in conventional linear regression model, respectively, yet the reduction in retinal venular caliber in Mendelian randomisation was insignificant. Considering the interaction between BP and hypertension, we further stratified our significant associations into non-hypertensive and hypertensive groups. The results remained consistently significant within two groups both in the observational model and the Mendelian randomisation model (supplementary material).

Our study has several strengths. All study participants followed the same standardised protocols and were examined at the same clinic, thus the measurements are comparable and vary consistently in terms of clinical performance. However, there was some potential bias to be considered in our analysis. First, we used the semi-automated vessel grading software for retinal vessel assessment; however, the manual input during the grading process might cause misclassification of the vessel parameters. Second, our sample size might not carry enough power to detect the true association between blood pressure and retinal vascular caliber, as most of the significant associations shown in conventional linear regression are attenuated in Mendelian randomisation. Third, even though our genetic risk scores were calculated based on individual weighted allele, there might still be a misspecification in our genetic mode, which requires further cross-validation validity. Unfortunately, we cannot identify any other cohort with BP GWAS data together with similar genetic background for one-third of our study participants (i.e., Malays). Therefore, such validation of our genetic risk scores approach may also be required in other samples of the same and different ethnicities to test for generalisation.

In summary, our findings showed weak association that genetically determined BP influences retinal arteriolar caliber but not retinal venular caliber to the same degree as the observed epidemiological associations. This suggests that elevated DBP and MAP and retinal arteriolar narrowing, but not retinal venular narrowing, are likely to represent a causal relationship. Changes in retinal venular caliber in accordance with elevated BP in conventional linear regression might be influenced by other major confounders such as dyslipdaemia or hyperglycaemia. Further elucidation of the role of retinal venules should provide insights into the pathophysiological mechanism of other chronic condition such as obesity or type 2 diabetes.

## Materials and Methods

### Study Population

The SEED Study is a population-based study, comprising three major ethnic groups in Singapore: Malays (the Singapore Malay Eye Study, 2004–2006), Indians (the Singapore Indian Eye Study, 2007–2009), and Chinese (the Singapore Chinese Eye Study, 2009–2011). All studies were conducted the same research team followed by the same protocol at the start in terms of recruitment and clinic examination. The detailed methodology of the SEED study has been published elsewhere, and the three ethnic group participants came to the same clinic and were examined by the same team with the same standardised protocol^28^,^29^. Briefly, SEED were launched with an invitation to all Singapore citizens or permanent residents aged 40–80+ years, residing in the South-East Singapore Ministry of Home Affairs database by using an age-stratified random sampling process. At the completion of recruitment, 3280 Singapore Malays, 3353 Singapore Chinese, and 3400 Singapore Indians had participated the clinical examination with a response rate of more than 75.6%. Participants who had both fundus photos available for retinal vascular caliber measurement, DNA sample for genetic analysis and were included in the present analysis. The final number of participants was 6528:2375 Malays, 2308 Indians, and 1845 Chinese.

This study was approved by both Singhealth Centralized Institutional Review Board and was conducted according to the tenets of the Declaration of Helsinki. Informed consents were obtained from participants prior to any examination.

### Clinical Examinations

#### Retinal photography and vessel assessment

SEED participants underwent a complete ocular examination, including a dilated fundus photography. Digital retinal photographs centered on the optic disc and macula were taken using a Canon 45° digital retinal camera (Model CR-DGi, Canon Inc). Measurement of retinal vascular caliber from the retinal photographs was performed at the Singapore Eye Research Institute, following a standardised protocol as described in previous reports in adults and children^2^,^12^,^28^,^29^. The computer imaging program (IVAN, University of Wisconsin, Madison, WI) was used to measure the caliber of all retinal arterioles and venules located in zone 0.5 to 1-disc diameter from the optic disc margin in the retinal photograph (zone B). By using the revised Knudtson-Parr-Hubbard formula to compute retinal vascular caliber, only the largest six arterioles and venules were used in calculating average vascular caliber, and estimates of the average diameters of arterioles and venules were summarised as CRAE and CRVE^30^. A single grader, masked to BP measurements and participant characteristics, performed all of the retinal vascular caliber measurements for this cohort. Intra-grader reliability was assessed in 70 randomly selected retinal photographs, and the intra-class correlation coefficient was 0.98 for CRAE and 0.99 for CRVE.

#### Blood pressure measurement

BP was taken with the participant seated and after five minutes of rest. SBP and DBP and pulse rate were measured with a digital automatic BP monitor (Dinamap model Pro Series DP110X-RW, 100V2; GE Medical Systems Information Technologies, Inc., USA) by following methods used in the Multi-Ethnic Study of Atherosclerosis (MESA)^28^,^29^. BP was measured on two occasions five minutes apart. If the BP differed by more than 10 mmHg systolic and 5 mmHg diastolic, a third measurement was made. The BP of the individual was then taken as the mean between the two closest readings. Mean arterial pressure (MAP) was calculated as one-third of SBP plus two-thirds of DBP.

#### Other measurement and questionnaire interview

The participant’s height was measured in centimeters using a wall-mounted measuring tape. Weight was measured in kilograms using a digital scale (SECA, model 7822321009; Vogel & Halke, Germany)^28^,^29^. Height was recorded to the nearest 1.0 mm while weight was recorded to the nearest 0.1 kg. BMI was calculated as weight divided by the height squared.

A 40 mL sample of non-fasting venous blood was collected to determine levels of serum lipids (total cholesterol, high density lipoprotein cholesterol, direct low density lipoprotein cholesterol), HbA1C, creatinine, and casual glucose. All serum biochemistry tests were sent to the National University Hospital Reference Laboratory for measurement on the same day. Blood was also collected for DNA extraction by using an automated DNA extraction technique at the Singapore Tissue Network. Extracted DNA samples were aliquoted and stored at −80°C.

A detailed interviewer-administered questionnaire was administered to collect relevant socio-demographic and medical information. The questionnaire was translated into English, Mandarin, Malay, and Tamil. Data collected included participants’ household income, education, occupation, current housing status, personal lifestyle factors (including past and current history on cigarette smoking and alcohol drinking), and individual systemic medical history such as hypertension and diabetes.

Presence of hypertension was defined as a participant had been diagnosed as hypertension or been taking anti-hypertensive medication before his/her enrolment in the SEED Study. Presence of diabetes was defined as a participant had been diagnosed as Type 1 or Type 2 diabetes mellitus, or had been taking diabetic medication before his/her enrolment in the SEED Study, or had random glucose level of 11.1 mmol/L and above.

### Genotyping

Participants were genotyped by using Human610-Quad BeadChip (Illumina, Inc. San Diego, CA), which included ~600,000 single nucleotide polymorphisms (SNPs). Stringent quality control filters were used to remove poorly performing samples and SNP markers. SNPs with a call-rate of <95%, MAF of <0.1%, or showing deviation from Equilibrium (P < 10^−6^) were removed. Routine quality control criteria on a per-sample basis were carried out, and poorly performing samples were removed from further analysis. The remaining samples were then subjected to biological relationship verification by using the principle of variability in allele sharing according to the degree of relationship. Identity-by-state information was derived using the PLINK software^31^. Pairs of individuals who showed evidence of cryptic relatedness were identified, and samples with the lower call rate were removed before performing principal component analysis (PCA). PCA was undertaken using smartPCA program (EIGENSTRAT software v4.2) accounting for spurious associations resulting from ancestral differences of individual SNPs^32^. Genotype data, which had undergone strict quality checks, were merged together and only SNPs shared by all three ethnic groups were used for analysis. This method and PCA figures have been described and shown in the published data in the same cohort^33^,^34^.

### Mendelian Randomisation Approach

#### Framework of Mendelian Randomisation

**Figure 1:**
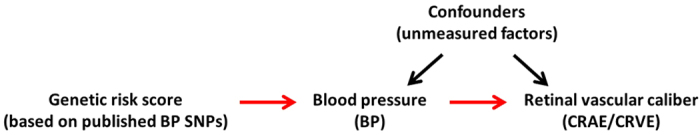
The theory of Mendelian randomisation by using BP genotype as an instrumental variable.

The framework of Mendelian randomisation by using genetic variants is described in Fig. 1. In this current analysis, we used BP genetic variants as IV for Mendelian randomisation analysis. In addition, we derived a GRS to combine effect from multiple known BP genetic variants (see more details below). The assumed validity of our Mendelian randomisation model with IV application is based on: *1*) BP genetic variants are associated with BP; *2*) BP genetic variants are independent of confounders for the association between BP and retinal vascular caliber in observational studies; and *3*) BP genetic variants are independent of retinal vascular caliber given BP level and the confounders.

#### Genetic Risk Scores of Blood Pressure

First, we identified 10 index SNPs from 10 genetic loci of SBP and DBP (Table 4) discovered from a few GWAS studies both in Europeans and East Asians^18^,^19^,^20^,^21^,^22^,^23^. All 10 genetic loci were identified with meta-analysis in Asian population while 4 of them were repeated in European population^23^. Due to the various genetic inheritances among Chinese, Malays, and Indians, we also included the 4 loci previously identified in Europeans to avoid selection bias. Candidate variants were searched based on a genomic region plus/minus 100 kb around each of the 10 index SNPs. Genotypes of SNPs were coded as 0, 1, and 2 for carrying 0, 1, and 2 copies of the risk alleles, respectively. Each SNP was tested for association with SBP, DBP, and MAP in the study participants using linear regression models. We then selected the most significantly associated SNP from each loci for each of the three BP phenotypes.

Second, we constructed SBP, DBP, and MAP-specific multi-locus GRSs for each individual by summing the number of risk alleles of the 10 selected SNPs, weighted by their estimated effect sizes (β from linear regression models) on each of the three BP phenotypes. This GRS method was adopted in previous literature^35^,^36^. The final GRSs of SBP, DBP, and MAP were treated as the IV of SBP, DBP, and MAP, respectively, for subsequent Mendelian randomisation analyses.

### Statistical Analysis

The associations between BP phenotypes and retinal vascular caliber were assessed in two ways: 1) conventional linear regression, and 2) Mendelian randomisation analysis. All analyses were performed using STATA (version 11.1, StataCorp, College Station, Texas). Two-tailed P values < 0.05 were considered significant.

In the conventional model, multiple linear regression was performed with retinal vascular caliber as outcome variables and BP as independent variable, adjusting for for age and gender and ethnicity (Model 1). Additional adjustment was made to account for potential confounders, including household income, BMI, total cholesterol level, blood glucose, blood creatinine level, presence of hypertension, presence of diabetes, cigarette smoking history, and alcohol drinking history (Model 2).

In the Mendelian randomisation model (Model 3), analysis was performed using a two-stage least squares approach with the STATA package ivreg2. The first stage was a linear regression assessing the association between BP GRSs and BP phenotypes (SBP, DBP, and MAP). The predicted value of BP phenotypes from the model was saved and used as an independent variable in the second stage, where the dependent variable was retinal vascular caliber (CRAE and CRVE). This effect size, or the IV estimate reflects an un-confounded effect of genetically determined BP level on retinal vascular caliber. Considering the potential pleiotropic effect in our Mendelian randomisation model, associations between BP GRSs and traditional confounders (e.g. cholesterol, serum glucose and serum creatinine) were further analysed.

## Additional Information

**How to cite this article**: Li, L.-J. *et al.* Assessing the Causality between Blood Pressure and Retinal Vascular Caliber through Mendelian Randomisation. *Sci. Rep.* **6**, 22031; doi: 10.1038/srep22031 (2016).

## Supplementary Material

Supplementary Information

## Figures and Tables

**Table 1:** Characteristics of Singapore Epidemiology of Eye Diseases (SEED) study participants.

Characteristics	Malay (n = 2308)	Indian (n = 2375)	Chinese (n = 1845)	Overall (n = 6528)
Age, years	58.5 (10.7)	57.6 (9.8)	58.3 (9.4)	58.1 (10.0)
Gender, Male	1147 (49.7)	1223 (51.5)	939 (50.9)	3309 (50.7)
Systolic blood pressure, mm Hg	147.07 (23.5)	135.5 (20.1)	135.5 (18.9)	139.6 (21.8)
Diastolic blood pressure, mm Hg	80.0 (11.3)	77.6 (10.3)	78.0 (9.7)	78.6 (10.6)
Mean arterial pressure, mm Hg	102.4 (14.0)	96.9 (12.2)	97.2 (11.3)	98.9 (12.9)
Body mass index, kg/m^2^	26.4 (5.1)	26.2 (4.8)	23.8 (3.5)	25.7 (4.7)
Serum glucose, mmol/L	6.7 (3.6)	7.1 (3.4)	6.3 (2.5)	6.8 (3.3)
Total cholesterol, mmol/L	5.6 (1.1)	5.2 (1.1)	5.5 (1.1)	5.4 (1.1)
Creatinine, mg/dl	92.8 (51.4)	77.2 (28.9)	76.3 (40.8)	82.4 (42.1)
CRAE, μm	139.5 (15.6)	143.3 (14.2)	140.8 (15.4)	141.3 (15.1)
CRVE, μm	219.4 (22.0)	208.5 (20.0)	208.4 (20.7)	212.3 (21.6)
Presence of hypertension*	1615 (70.0)	1402 (59.0)	1064 (57.7)	4081 (62.5)
Presence of diabetes†	531 (23.0)	783 (33.0)	232 (12.6)	1546 (23.4)
Smoking history, Yes‡	915 (39.7)	649 (27.3)	489 (26.5)	2053 (31.4)
Alcohol drinking, Yes§	34 (1.5)	314 (13.2)	216 (11.7)	564 (8.6)
Household income, <1000 SGD/month	1542 (66.8)	1137 (47.9)	817 (44.3)	3496 (52.7)

Data presented are mean (standard deviation) for continuous variables and n (%) for categorical variables.

Abbreviations: CRAE, central retinal arteriolar equivalent; CRVE, central retinal venular equivalent; SGD, Singapore Dollar.

^*^Presence of hypertension: defined as a participant had been diagnosed as hypertension or been taking anti-hypertensive medication before his/her enrolment in the SEED Study.

^†^Presence of diabetes: defined as a participant had been diagnosed as Type 1 or Type 2 diabetes Mellitus, or had been taking diabetic medication before his/her enrolment in the SEED Study, or had random glucose level of 11.1 mmol/L and above.

^‡^Smoking history includes past and current smoking.

^§^Alcohol drinking includes past and current alcohol drinking.

**Table 2:** Assessing the association between blood pressure and retinal vascular caliber by using conventional multiple linear regression model vs. Mendelian randomisation analysis.

Blood Pressure	CRAE		CRVE	
	β§ (95% CI)	P	β§ (95% CI)	P
**Systolic blood pressure, per 10** **mm Hg ↑**				
Model 1*	−1.4 (−1.6, −1.3)	<0.001	−0.5 (−0.7, −0.2)	<0.001
Model 2†	−1.4 (−1.6, −1.2)	<0.001	−0.6 (−0.9, −0.4)	<0.001
Model 3: Mendelian randomisation‡	−1.3 (−2.8, 0.3)	0.10	−1.0 (−3.7, 1.7)	0.46
**Diastolic blood pressure, per 10** **mm Hg ↑**				
Model 1*	−3.1 (−3.5, −2.8)	<0.001	−0.6 (−1.1, −0.1)	0.03
Model 2†	−3.0 (−3.4, −2.6)	<0.001	−0.7 (−1.2, −0.2)	<0.01
Model 3: Mendelian randomisation‡	−4.5 (−7.9, −1.0)	0.01	−2.3 (−7.4, 2.7)	0.36
**Mean arterial pressure, per 10** **mm Hg ↑**				
Model 1*	−2.6 (−2.9, −2.3)	<0.001	−0.6 (−1.1, −0.2)	<0.01
Model 2†	−2.6 (−2.9, −2.3)	<0.001	−0.9 (−1.3, −0.5)	<0.001
Model 3: Mendelian randomisation‡	−2.6 (−5.2, 0.01)	0.05	0.2 (−3.6, 4.0)	0.92

Abbreviations: CRAE, central retinal arteriolar equivalent; CRVE, central retinal venular equivalent; CI, Confidence Interval.

^*^Model 1: adjusting for age and gender, and ethnicity.

^†^Model 2: adjusting for age, gender, ethnicity, household income, body mass index, total cholesterol, blood glucose, blood creatinine level, anti-hypertension medication, diabetes history, smoking history and alcohol drinking history.

^‡^Model 3: adjusting for age, gender and the first 5 genetic principal components.

^§^β: effect size or correlation coefficient of the linear regression model.

**Table 3:** Assessing the associations between blood pressure genetic risk scores and traditional biomarkers as potential confounders for elevated blood pressure.

Traditional confounders for elevated blood pressure	SBP GRS, per 1 score increase		DBP GRS, per 1 score increase		MAP GRS, per 1 score increase	
	β, SE	P	β, SE	P	β, SE	P
Total cholesterol, mmol/L	0.01, 0.02	0.62	−0.02, 0.01	0.19	0.001, 0.01	0.94
LDL, mmol/L	0.05, 0.03	0.06	−0.002, 0.01	0.90	0.006	0.50
Serum glucose, mmol/L	0.02, 0.01	0.06	0.004, 0.004	0.28	0.30	0.35
Serum creatinine, mg/dl	0.04, 0.06	0.53	0.01, 0.03	0.63	−0.15	0.72

Abbreviations: SBP, systolic blood pressure; DBP, diastolic blood pressure; MAP, mean arterial pressure; GRS, genetic risk score; high-density lipoprotein; LDL, low-density lipoprotein; HbA1C, glycated haemoglobin; β, beta; SE, standard error.

**Table 4:** Blood pressure single nucleotide polymorphisms (SNPs) selected for this study.

no	Chromoso me	SNP*	Nearby Gene	Starting BP	Ending BP	Ethnicity
1	1	rs17030613	*ST7L-CAPZA1*	112767664	113115764	Asians
2	1	rs880315	*CASZ1*	10596666	10956733	Europeans
3	2	rs16849225	*FIGN-GRB14*	164074308	165286606	Asians
4	4	rs16998073	*FGF5*	81084091	81312171	Europeans
5	4	rs6825911	*ENPEP*	111516678	111803942	Asians
6	5	rs1173766	NPR3	32610743	32904778	Asians
7	10	rs11191548	*CNNM2-NT5C2-CYP17A1*	104490288	105053064	Europeans
8	12	rs11066280	*RPL6-PTPN11-ALDH2*	112104691	113024727	Asians
9	12	rs35444	*TBX3*	115008059	115652687	Asians
10	12	rs17249754	*ATP2B1*	89881826	90160836	Europeans

Abbreviations: BP, blood pressure.

^*^Candidate variants were searched based on a genomic region plus/minus 100 kb around each of the 10 index SNPs, based on a series of published GWAS studies either in European or Asian population.
