# Supplementary material for: Assessing the Causality between Blood Pressure and Retinal Vascular Caliber through Mendelian Randomisation
Source: Sci Rep. 2016 Feb 25;6:22031. doi: 10.1038/srep22031 (PMC4766565; doi:10.1038/srep22031)
Supplement: Supplementary Information [file srep22031-s1.doc]

**Assessing the Causality between Blood Pressure and Retinal Vascular Caliber through Mendelian Randomisation**

Ling-Jun Li,^1,2^ Jiemin Liao^1,3^, Carol Yim-Lui Cheung^1,2,4^, M. Kamran Ikram^1,2^, Tai E Shyong^3^, Tien-Yin Wong^1,2^, Ching-Yu Cheng^1,2,5^

^1^ Singapore Eye Research Institute, Singapore National Eye Centre, Singapore;

^2^ Duke-NUS Graduate Medical School, Singapore

^3^ Department of Medicine, National University of Singapore and National University Health System, Singapore

^4^ Department of Ophthalmology and Visual Sciences, The Chinese University of Hong Kong, Hong Kong.

^5^ Department of Ophthalmology, National University of Singapore and National University Health System, Singapore

Short title: Causality of blood pressure and retinal vessels

Correspondence author: A/Prof. Ching-Yu Cheng, 20 College Road, The Academia, Level 6, Discovery Tower, Singapore 169856, Tel: +65 65767277, Fax: +65 6225 2568 , Email: chingyu.cheng@duke-nus.edu.sg

**Supplementary Table: Assessing the association between blood pressure and retinal vascular caliber by using conventional multiple linear regression model vs. Mendelian randomisation analysis.**

| **Blood Pressure** | **CRAE, µm**  **β**^‡^  **(95% CI)** | | **CRVE, µm**  **β**^‡^  **(95% CI)** | |
| --- | --- | --- | --- | --- |
|  | **Presence of hypertension** | **Non-hypertension** | **Presence of hypertension** | **Non-hypertension** |
| **Systolic blood pressure, per 10 mm Hg↑** |  |  |  |  |
| Multivariate linear model* | -1.00 (-1.26, -0.75) | -2.57 (-3.09, -2.04) | -0.56 (-0.92, -0.20) | -1.41 (-2.16, -0.66) |
| P value | <0.001 | <0.001 | 0.002 | <0.001 |
| Mendelian randomisation^†^ | -4.51 (-11.80, 2.78) | -0.19 (-2.50, 2.12) | 3.53 (-6.94, 14.01) | 1.85 (-1.50, 5.20) |
| P value | 0.225 | 0.871 | 0.508 | 0.279 |
| **Diastolic blood pressure, per 10 mm Hg↑** |  |  |  |  |
| Multivariate linear model* | -3.39 (-4.19, -2.58) | -2.43 (-2.90, -1.96) | -0.62 (-1.29, 0.04) | -0.79 (-1.92, 0.35) |
| P value | <0.001 | <0.001 | 0.066 | 0.176 |
| Mendelian randomisation^†^ | -4.42 (-15.67, 6.82) | -3.84 (-8.31, 0.63) | -4.06 (-21.16, 13.04) | -1.77 (-8.24, 4.69) |
| P value | 0.441 | 0.092 | 0.641 | 0.591 |
| **Mean arterial pressure, per 10 mm Hg↑** |  |  |  |  |
| Multivariate linear model* | -2.05 (-2.46, -1.65) | -3.86 (-4.63, -3.09) | -0.78 (-1.36, -0.21) | -1.47 (-2.55, -0.38) |
| P value | <0.001 | <0.001 | 0.008 | 0.008 |
| Mendelian randomisation^†^ | -4.02 (-13.64, 5.61) | -1.58 (-5.54, 2.38) | 4.40 (-9,77, 18.57) | -0.08 (-5.91, 5.76) |
| P value | 0.413 | 0.434 | 0.543 | 0.979 |

Abbreviations: CRAE, central retinal arteriolar equivalent; CRVE, central retinal venular equivalent; CI, Confidence Interval.

^*^ Multivariate linear model: adjusting for age, gender, ethnicity, household income, BMI, total cholesterol, blood glucose, blood creatinine level, anti-hypertension medication, diabetes history, smoking history and alcohol drinking history.

^†^ Mendelian Randomization model: adjusting for age, gender and the first 5 genetic principal components.

^‡^ β: effect size or correlation coefficient of the linear regression model.
